# Insights From the National Inpatient Sample (2016–2019) on Laryngeal Cancer Incidence and Trends

**DOI:** 10.7759/cureus.61660

**Published:** 2024-06-04

**Authors:** Ebraheem Albazee, Abdullah M Alharran, Mooza M Alzayed, Yousef M Alharran, Fajer B Alyaqout, Ahmed Almutairi, Ahmed Abu-Zaid

**Affiliations:** 1 Department of Internship, Kuwait Institute for Medical Specializations, Kuwait City, KWT; 2 College of Medicine, Arabian Gulf University, Manama, BHR; 3 Faculty of Medicine, Alexandria University, Alexandria, EGY; 4 College of Medicine, Alfaisal University, Riyadh, SAU

**Keywords:** trends, medicare, national inpatient sample, epidemiology, laryngeal cancer

## Abstract

Background: Laryngeal cancer has a significant impact on speech, swallowing, and quality of life. This study aims to analyze laryngeal cancer trends using the National Inpatient Sample (NIS) database, providing insights into its epidemiology.

Methods: Data from the NIS database was analyzed for a cohort of 14,282 laryngeal cancer cases from 2016 to 2019. Baseline characteristics and demographic parameters, including primary expected payer, age groups, hospital types, and geographic regions, were examined. Descriptive statistics and trend analysis were conducted.

Results: The cohort showed consistent annual case numbers (range: 3739-3948). The highest case numbers were in the 40-64 age group (average 1998 cases/year), followed by the 65-80 age group (average 1473 cases/year). Medicare was the most common primary expected payer, followed by Medicaid, private insurance, self-pay, and no charge. The cohort was roughly three times more skewed toward males, with an average of 2936 male cases per year compared to 885 female cases. Notable trends included significant positive correlations with time for urban teaching hospitals, the South region, older age group (65-80 years), and Asian or Pacific Islander individuals. However, the overall correlation between case numbers and time was not statistically significant. The primary expected payer and deaths exhibited moderate correlations with time but did not reach statistical significance.

Conclusion: This study provides insights into the baseline characteristics and trends in laryngeal cancer incidence. The observed demographic shifts highlight the need for further investigation into underlying factors influencing case distribution. Understanding these trends can guide targeted interventions for prevention, early detection, and treatment of laryngeal cancer.

## Introduction

Laryngeal cancer is a significant public health concern, characterized by the development of malignant tumors in the larynx [1,2]. It poses substantial challenges to patients and healthcare systems due to its impact on speech, swallowing, and overall quality of life [3]. Understanding the incidences, trends, and characteristics of laryngeal cancer is crucial for effective healthcare planning, resource allocation, and the development of targeted interventions.

Laryngeal cancer has been strongly linked to certain risk factors, including tobacco and alcohol use, exposure to occupational hazards such as asbestos or chemicals, and infection with high-risk types of human papillomavirus (HPV) [4-8]. These risk factors can vary in prevalence among different populations and geographic regions, potentially contributing to variations in the incidence of laryngeal cancer [2,7,9-11]. By assessing the National Inpatient Sample (NIS) data, we can investigate the association between these risk factors and the development of laryngeal cancer, helping to inform targeted prevention and public health strategies.

In this study, we aim to analyze the trends in laryngeal cancer incidence using data from the NIS, one of the largest all-payer inpatient healthcare databases in the United States [12]. The specific objectives were: (i) to describe the baseline characteristics of individuals with laryngeal cancer from 2016 to 2019, and (ii) to analyze the laryngeal cancer cases over time and any associations between baseline characteristics and time. By examining a comprehensive dataset capturing information from millions of hospital stays, the NIS provides a representative sample of the United States population and allows for a robust assessment of the national trends in laryngeal cancer. Ultimately, the goal of this study is to enhance our understanding of laryngeal cancer trends using the NIS data and to inform evidence-based interventions and policies that aim to reduce the burden of laryngeal cancer on individuals and society.

## Materials and methods

This study employed a retrospective observational design, utilizing data from the NIS database, the Healthcare Cost and Utilization Project (HCUP), and the Agency for Healthcare Research and Quality. The study focused on individuals diagnosed with laryngeal cancer between the years 2016 and 2019. Cohort selection was based on relevant International Classification of Diseases, Tenth Revision, and Clinical Modification (ICD-10-CM) codes specifically indicating malignant laryngeal cancer (C32). This general code (C32) includes several subcodes, namely malignant neoplasms of glottis (C32.0), supraglottis (C32.1), subglottis (C32.2), laryngeal cartilage (C32.3), overlapping sites of larynx (C32.8), and unspecific larynx (C32.9). Data from the NIS database was extracted, including demographic variables, hospital characteristics, and in-hospital mortality. The demographic variables included age (0-18, 18-49, 40-65, 65-80, and >80 years), gender (male and female), race (White, Black, Hispanic, Asian or Pacific Islander, Native American, and other), zip income quartile (1st-25th, 26th-50th, 51st-75th, and 76th-100th), and primary expected payer (Medicare, Medicaid, private insurance, self-pay, and no charge). The hospital characteristics included location/teaching status (rural, urban nonteaching, and urban teaching) and geographic region (Northeast, Midwest or North Central, South, and West).

STATA software, version 18, was used for data analysis. Descriptive statistics were used to examine the baseline characteristics of the cohort, including the number of cases, age distribution, gender distribution, primary expected payer distribution, hospital types, and geographic regions. Trends in these variables over the study period were analyzed. Linear regression analysis was conducted to identify any associations between the number of laryngeal cancer cases and time, as well as the relationship between demographic/hospital parameters and time. Data were presented as correlation coefficients and 95% confidence intervals. Statistical significance was established as a p-value less than 0.05.

Ethical approval is not required as the publicly available data are anonymous and de-identified.

## Results

Table 1 provides an overview of the baseline characteristics of a cohort of individuals with laryngeal cancer from 2016 to 2019. The cohort consisted of a total of 14,282 cases over the 4-year period. The number of laryngeal cancer cases remained relatively consistent each year, ranging from 3739 cases in 2016 to 3948 cases in 2019. Within the cohort, 727 individuals unfortunately passed away during this time, with the number of deaths varying slightly each year. The cohort was stratified by age category, with most cases falling within the 40-64 age group (average 1998 cases per year), followed by the 65-80 age group (average 1473 cases per year). Smaller numbers of cases were observed in the 0-17, 18-39, and >80 age groups. Regarding the primary expected payer for medical expenses, Medicare was the most common payer, covering the treatment costs for a substantial portion of the cohort each year. Medicaid and private insurance were the next most prevalent payers, followed by self-pay and no-charge (indicating cases where no payment was required). In terms of gender, the cohort was roughly three times more skewed toward males, with an average of 2936 male cases per year compared to 885 female cases. The distribution of cases according to zip income quartile showed a higher concentration in the 1st-25th quartile, followed by the 26th-50th, 51st-75th, and 76th-100th quartiles. The cohort was primarily treated in urban teaching hospitals, which accounted for the majority of cases each year. Rural and urban nonteaching hospitals treated smaller proportions of the cohort. Geographically, the highest number of cases was observed in the South region, followed by the Midwest or North Central, West, and Northeast regions.

**Table 1:** The baseline characteristics of the analyzed cohort. The data are presented as numbers (percentages).

Year	2016	2017	2018	2019
Number of laryngeal cancer cases	3739	3720	3875	3948
In-hospital mortality	201 (5.38)	179 (4.81)	170 (4.39)	177 (4.48)
Age Category (years)				
0-17	0 (0)	3 (0.08)	8 (0.21)	3 (0.08)
18-39	25 (0.67)	34 (0.91)	32 (0.83)	33 (0.84)
40-64	2011 (53.78)	1958 (52.63)	2006 (51.77)	2017 (51.09)
65-80	1417 (37.9)	1443 (38.79)	1486 (38.35)	1546 (39.16)
>80	286 (7.65)	282 (7.58)	343 (8.85)	349 (8.84)
Primary Expected Payer				
Medicare	2008 (53.7)	2032 (54.62)	2158 (55.69)	2171 (54.99)
Medicaid	791 (21.16)	773 (20.78)	761 (19.64)	790 (20.01)
Private insurance	686 (18.35)	684 (18.39)	720 (18.58)	706 (17.88)
Self-pay	101 (2.7)	89 (2.39)	105 (2.71)	121 (3.06)
No-charge	6 (0.16)	6 (0.16)	5 (0.13)	9 (0.23)
Female gender	890 (23.8)	858 (23.06)	919 (23.72)	873 (22.11)
Zip income Quartile				
1st-25th	1441 (38.54)	1406 (37.8)	1395 (36)	1464 (37.08)
26th-50^th^	961 (25.7)	998 (26.83)	1098 (28.34)	990 (25.08)
51st-75^th^	739 (19.76)	733 (19.7)	795 (20.52)	831 (21.05)
76th-100^th^	535 (14.31)	531 (14.27)	515 (13.29)	587 (14.87)
Location/teaching status of hospital				
Rural	284 (7.6)	275 (7.39)	262 (6.76)	275 (6.97)
Urban nonteaching	675 (18.05)	555 (14.92)	548 (14.14)	490 (12.41)
Urban teaching	2780 (74.35)	2890 (77.69)	3065 (79.1)	3183 (80.62)
Hospital Region				
Northeast	749 (20.03)	659 (17.72)	710 (18.32)	757 (19.17)
Midwest or North Central	959 (25.65)	956 (25.7)	1011 (26.09)	919 (23.28)
South	1534 (41.03)	1597 (42.93)	1660 (42.84)	1746 (44.22)
West	497 (13.29)	508 (13.66)	494 (12.75)	526 (13.32)
Race				
White	0 (0)	3 (0.08)	8 (0.21)	3 (0.08)
Black	22 (0.59)	27 (0.73)	30 (0.77)	29 (0.73)
Hispanic	1904 (50.92)	1845 (49.6)	1862 (48.05)	1880 (47.62)
Asian or Pacific Islander	1480 (39.58)	1515 (40.73)	1584 (40.88)	1646 (41.69)
Native American	332 (8.88)	330 (8.87)	391 (10.09)	390 (9.88)
Other	2568 (68.68)	2594 (69.73)	2703 (69.75)	2784 (70.52)

**Figure 1:**
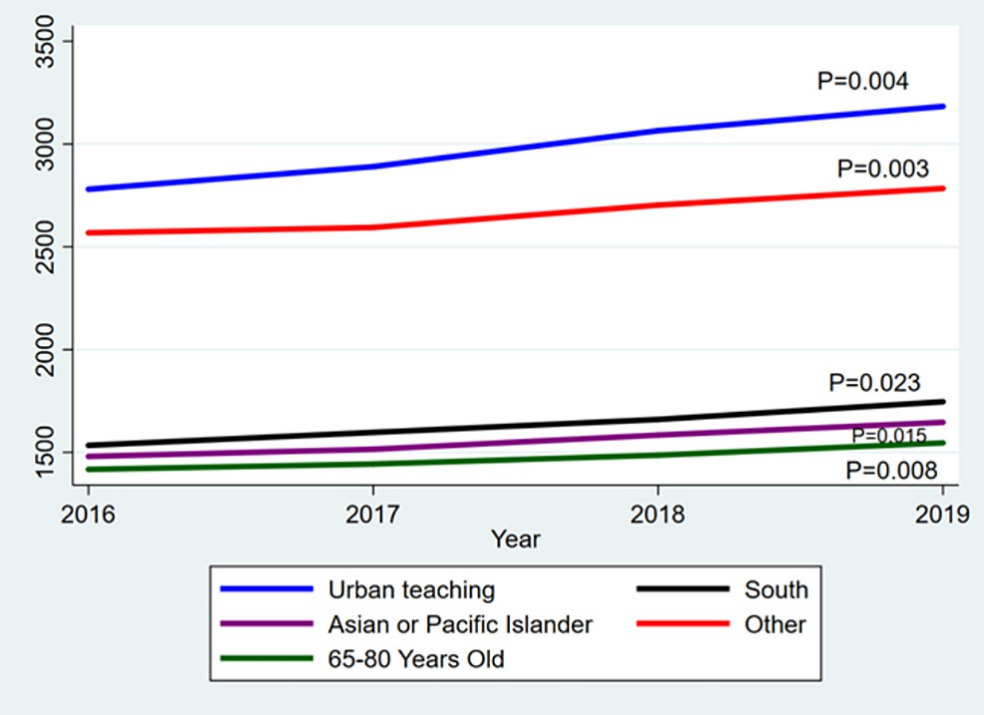
The significant demographic parameters for a cohort of individuals diagnosed with laryngeal cancer.

Table 2 presents notable findings concerning the trends in demographic parameters for a cohort of individuals diagnosed with laryngeal cancer. Several variables displayed statistically significant correlations with time. First, urban teaching hospitals exhibited a strong positive correlation, indicating a significant increase in the number of cases treated within these institutions over the studied period. Similarly, the South region demonstrated a noteworthy positive correlation, suggesting a substantial rise in the incidence of laryngeal cancer within that specific geographic area. Additionally, Asian or Pacific Islander individuals and the 65-80 age group displayed a significant positive correlation, indicating an upward trend in cases among these racial and age groups. These significant outcomes shed light on specific shifts in the distribution of laryngeal cancer cases, emphasizing the need for further investigation into the underlying factors driving these changes. On the other hand, the overall correlation between the number of laryngeal cancer cases and time was moderate but not statistically significant. Likewise, the primary expected payer, encompassing Medicare, Medicaid, private insurance, self-pay, and no charge, did not exhibit significant correlations with time. Although the variable for deaths exhibited a slight negative correlation, suggesting a potential decrease over the years, this association did not reach statistical significance. Figure 1 illustrates the significant trends observed among the analyzed variables.

**Table 2:** Trends of demographic parameters over the years (2016-2019). *Statistical significance at p-value <0.05.

Variable	Correlation Coefficient	95% Lower Limit	95% Upper Limit	p-value
Laryngeal cancer cases	78.2	-21.7179	178.1179	0.078
Medicare	61.5	-4.33902	127.339	0.057
Medicaid	-1.5	-35.2011	32.20106	0.866
Private insurance	9.6	-18.3904	37.5904	0.278
Self-pay	7.6	-13.2668	28.46676	0.258
No charge	0.8	-2.4768	4.076803	0.404
In-hospital mortality	-8.1	-27.8501	11.65007	0.22
Female gender	1	-60.5896	62.58958	0.951
1st-25th zip income quartile	5.8	-66.9746	78.57464	0.764
26th-50th zip income quartile	18.7	-109.864	147.2644	0.595
51st-75th zip income quartile	33.8	-6.62662	74.22662	0.069
76th-100th zip income quartile	14	-45.9908	73.99077	0.421
Rural hospital	-4	-21.5303	13.53034	0.43
Urban nonteaching hospital	-56.2	-121.535	9.135183	0.066
Urban teaching hospital	138.4	100.8854	175.9146	0.004*
Northeast region	7.5	-95.6157	110.6157	0.784
Midwest or North Central region	-6.5	-93.4253	80.4253	0.778
South region	69.9	52.75945	87.04055	0.003*
West region	7.3	-18.5983	33.19825	0.349
White race	1.4	-5.15361	7.953605	0.455
Black race	2.4	-1.72696	6.52696	0.129
Hispanic race	-5.5	-62.6379	51.63787	0.719
Asian or Pacific Islander race	56.7	34.49648	78.90352	0.008*
Native American race	23.5	-14.5365	61.53652	0.117
Other race	75.7	25.29648	126.1035	0.023*
0-17 age group (years)	1.4	-5.153605	7.953605	0.455
18-39 age group (years)	2.2	-4.711084	9.111084	0.304
40-64 age group (years)	6.6	-53.88557	67.08557	0.685
65-80 age group (years)	43	19.86949	66.13051	0.015*
>80 age group (years)	25	-12.13765	62.13765	0.101

## Discussion

This study explored patient characteristics associated with laryngeal cancer, such as age, gender, race/ethnicity, and socioeconomic factors using the NIS database. Understanding how these factors interact with the incidence of laryngeal cancer can provide insights into potential disparities in disease burden, access to healthcare, and treatment outcomes. By identifying high-risk populations and vulnerable groups, healthcare professionals, and policymakers can develop tailored interventions to improve early detection, diagnosis, and treatment outcomes for laryngeal cancer.

The consistent number of laryngeal cancer cases observed each year indicates a stable incidence rate during the study period. The predominance of Medicare as the primary expected payer reflects the higher prevalence of this disease among older adults, who are more likely to be covered by Medicare.

Laryngeal cancer, particularly squamous cell carcinoma (SCC), is influenced by a range of risk factors encompassing lifestyle choices, environmental exposures, infections, and genetic predispositions [13,14]. The incidence of laryngeal cancer is notably higher in older age groups and males, primarily due to the cumulative effects of these risk factors over time [13], which is consistent with our findings. The most significant risk factor is smoking and tobacco use, as the carcinogens in tobacco can cause DNA mutations in laryngeal cells, with the risk increasing with prolonged and intense smoking [14,15]. Heavy alcohol use also significantly raises the risk, especially when combined with smoking [14,15]. Infections from viruses like HPV and Epstein-Barr virus can integrate into the host genome, disrupting normal cellular functions and leading to malignancies [13-15]. Occupational exposures to chemicals such as asbestos, sulfuric acid, and paint fumes cause chronic irritation and inflammation of the larynx, promoting cancer development [13-15]. Gastroesophageal reflux disease is another risk factor, as chronic acid reflux can damage the laryngeal lining, leading to cellular changes and increased cancer risk [13-15]. Genetic susceptibility plays a role as well, with certain genetic polymorphisms, such as those in the *DIAPH2*, *PTPRD*, and *HIC1* genes, being associated with an increased risk of laryngeal cancer [16]. Additionally, a poor diet, particularly a low intake of fruits and vegetables, can impair the body’s ability to repair DNA damage and maintain healthy cellular functions, further increasing cancer risk [14,15].

A recent review included 99 cases of laryngeal cancer in patients younger than 30 years. The data was obtained from the National Cancer Institute's Surveillance, Epidemiology, and End Results (SEER) Program [17]. Most of the patients were white and aged 25 to 29 years, with slightly more females than males. The most common type of cancer was SCC, primarily affecting the glottis. The 5-year relative survival rate was lowest among those aged 15 to 19 years (60.1%), while those aged 20 to 24 and 25 to 29 years had higher survival rates (87.7% and 87.4%, respectively) [17]. The causes of SCC in children and adolescents remain uncertain, but in adults, smoking, drinking, and poor oral hygiene are considered risk factors. Laryngeal cancer in young people has been associated with malignant degeneration of papillomas and complications of radiotherapy. Infection with HIV may accelerate SCC development in patients with significant risk factors by impairing immune surveillance mechanisms [17].

Laryngeal carcinoma in young adults was studied in a retrospective chart review of 29 patients under 40 years old to determine optimal treatment and survival outcomes. The study found that laryngeal SCC behaves similarly in young adults compared to older adults. Patients treated in the organ preservation era had higher 2-year laryngectomy-free survival rates without significant changes in overall survival [18].

A study comparing younger and older patients with laryngeal cancer found that the younger group had a higher proportion of female patients, more cases of glottic involvement, and a lower incidence of distant metastasis [19]. The 5-year overall survival and cancer-specific survival rates were significantly better in younger patients compared to older patients. Advanced tumor stage, nodal involvement, and distant metastasis were negative prognostic factors in younger patients. Treatment with surgery and/or radiation therapy resulted in excellent outcomes for all stages of the disease, and radical radiation therapy was as effective as total laryngectomy in locally advanced laryngeal SCC among young patients [19].

The significant representation of cases in urban teaching hospitals highlights their importance in diagnosing and treating laryngeal cancer. The concentration of cases in these institutions suggests that they play a critical role in managing this disease and indicates the presence of specialized facilities and experienced healthcare professionals. Conversely, the smaller proportions of cases observed in rural and urban nonteaching hospitals may reflect limitations in resources and expertise for managing laryngeal cancer in these settings. These findings contradict the previous study conducted by Zuniga and Lango, which reported that rural populations have a higher risk of developing laryngeal cancer [20]. Besides, Pagedar et al. reported a declining trend of laryngeal cancer cases in urban areas compared to rural areas [21].

Geographically, the highest number of cases was observed in the South region, followed by the Midwest or North Central, West, and Northeast regions. This regional disparity in the incidence of laryngeal cancer may be attributed to variations in risk factors, access to healthcare services, socioeconomic disparities, and environmental exposures. Nonetheless, future research is warranted to delineate the underlying risk factors for this observation.

Another notable finding is the significant positive correlation among Asian or Pacific Islander individuals, indicating an increasing trend in laryngeal cancer cases within this racial group. This observation highlights the need for further investigation into potential cultural, genetic, and environmental factors that may contribute to the rising incidence of laryngeal cancer among Asian or Pacific Islander populations. Khosla et al. discovered that individuals from Asian diasporas have a higher risk of developing certain types of head and neck SCCs (HNSCCs) [22].

In a recent study, authors also found that South Asian individuals had a higher rate of head and neck cancer (HNC) diagnosis. After adjusting for various factors, the hazard ratio (HR) for HNC diagnosis among South Asian individuals was 1.29 (95% CI: 1.14-1.45) [23]. The rising trend in the number of laryngeal cancer cases among Asian or Pacific individuals can be linked to the growing presence of this racial group within the American population [24].

This study has several limitations that should be considered when interpreting the results. First, the use of data from the NIS database introduces potential limitations related to data accuracy and completeness, as some demographic features were missing. The coding practices and documentation in the original hospital records may vary, leading to possible coding errors or misclassification of cases. This could impact the validity of the findings and limit the generalizability of the results to other populations or healthcare settings. Second, the study focused on laryngeal cancer cases within a specific time frame (2016-2019), which may not capture long-term trends or changes in the incidence and characteristics of the disease. A longer observation period would provide a more comprehensive understanding of the temporal patterns and allow for the assessment of any emerging trends or variations over time. Lastly, the analysis was limited to the variables available in the NIS database. Important factors that could influence laryngeal cancer incidence, such as lifestyle factors, occupational exposures, and genetic predisposition, were not considered. The absence of these variables may limit the ability to fully explore the complex factors contributing to laryngeal cancer trends.

## Conclusions

Notable trends were identified in demographic parameters, including an increase in cases treated in urban teaching hospitals, in the South region, and among Asian or Pacific Islander individuals and older age groups. These trends call for further investigation into the underlying factors influencing these patterns. Despite the limitations, the findings of this study have implications for healthcare planning, resource allocation, and the development of targeted interventions to improve prevention, early detection, and treatment strategies for laryngeal cancer.

## References

[1] Nocini R, Molteni G, Mattiuzzi C, Lippi G (2020). Updates on larynx cancer epidemiology. Chin J Cancer Res.

[2] Jones TM, De M, Foran B, Harrington K, Mortimore S (2016). Laryngeal cancer: United Kingdom National Multidisciplinary guidelines. J Laryngol Otol.

[3] Van den Bogaert W, Ostyn F, van der Schueren E (1983). The different clinical presentation, behaviour and prognosis of carcinomas originating in the epilarynx and the lower supraglottis. Radiother Oncol.

[4] West R (2017). Tobacco smoking: Health impact, prevalence, correlates and interventions. Psychol Health.

[5] Dabirmoghaddam P, Karimian Taheri A, Ghazavi H, Ebrahimnejad S, Karimian Z (2016). Does opium dependency affect the pattern of involvement in laryngeal cancer?. Iran J Otorhinolaryngol.

[6] Altieri A, Garavello W, Bosetti C, Gallus S, La Vecchia C (2005). Alcohol consumption and risk of laryngeal cancer. Oral Oncol.

[7] Nikakhlagh S, Saki N, Shoar MH, Sartipipor A, Saki S (2012). Incidence of etiologic factors in squamous cell carcinoma of head and neck in ahvaz. Iran J Otorhinolaryngol.

[8] Stumbrytė-Kaminskienė A, Gudlevičienė Ž, Dabkevičienė D, Mackevičienė I (2020). Combined Effect of HPV and Several Gene SNPs in Laryngeal Cancer. Medicina (Kaunas).

[9] Chatenoud L, Garavello W, Pagan E (2016). Laryngeal cancer mortality trends in European countries. Int J Cancer.

[10] Menach OP, Patel A, Oburra HO (2014). Demography and histologic pattern of laryngeal squamous cell carcinoma in kenya. Int J Otolaryngol.

[11] Hoffman HT, Porter K, Karnell LH (2006). Laryngeal cancer in the United States: Changes in demographics, patterns of care, and survival. Laryngoscope.

[12] (2024). HCUP National Inpatient Sample (NIS). Healthcare Cost and Utilization Project (HCUP). http://www.hcup-us.ahrq.gov/nisoverview.jsp.

[13] Lin C, Cheng W, Liu X, Li H, Song Y (2022). The global, regional, national burden of laryngeal cancer and its attributable risk factors (1990-2019) and predictions to 2035. Eur J Cancer Care (Engl).

[14] Liberale C, Soloperto D, Marchioni A, Monzani D, Sacchetto L (2023). Updates on larynx cancer: Risk factors and oncogenesis. Int J Mol Sci.

[15] Kim DH, Kim SW, Han JS, Kim GJ, Basurrah MA, Hwang SH (2023). The prognostic utilities of various risk factors for laryngeal squamous cell carcinoma: A systematic review and meta-analysis. Medicina (Kaunas).

[16] Śnit M, Misiołek M, Ścierski W, Koniewska A, Stryjewska-Makuch G, Okła S, Grzeszczak W (2021). DIAPH2, PTPRD and HIC1 gene polymorphisms and laryngeal cancer risk. Int J Environ Res Public Health.

[17] Rutt AL, Hawkshaw MJ, Sataloff RT (2010). Laryngeal cancer in patients younger than 30 years: A review of 99 cases. Ear Nose Throat J.

[18] Nachalon Y, Cohen O, Alkan U, Shvero J, Popovtzer A (2017). Characteristics and outcome of laryngeal squamous cell carcinoma in young adults. Oncol Lett.

[19] Singh B, Alfonso A, Sabin S, Poluri A, Shaha AR, Sundaram K, Lucente FE (2000). Outcome differences in younger and older patients with laryngeal cancer: A retrospective case-control study. Am J Otolaryngol.

[20] Zuniga SA, Lango MN (2018). Effect of rural and urban geography on larynx cancer incidence and survival. Laryngoscope.

[21] Pagedar NA, Kahl AR, Tasche KK, Seaman AT, Christensen AJ, Howren MB, Charlton ME (2019). Incidence trends for upper aerodigestive tract cancers in rural United States counties. Head Neck.

[22] Khosla S, Hershow RC, Freels S, Jefferson GD, Davis FG, Peterson CE (2021). Head and neck squamous cell carcinomas among males of the three largest Asian diasporas in the US, 2004-2013. Cancer Epidemiol.

[23] Noel CW, Sutradhar R, Li Q (2020). Association of immigration status and Chinese and South Asian ethnicity with incidence of head and neck cancer. JAMA Otolaryngol Head Neck Surg.

[24] Chen MS, Lee RJ, Madan RA, Ta Park V, Shinagawa SM, Sun T, Gomez SL (2022). Charting a path towards Asian American cancer health equity: A way forward. J Natl Cancer Inst.

